# COVID-19 and the reimaging of compassionate release

**DOI:** 10.1108/IJPH-08-2021-0072

**Published:** 2022-06-24

**Authors:** Jennifer E. James, Meghan Foe, Riya Desai, Apoorva Rangan, Mary Price

**Affiliations:** Institute for Health and Aging, University of California San Francisco, San Francisco, California, USA.; School of Medicine, University of California San Francisco, San Francisco, California, USA.; Joint Medical Program at the University of California, San Francisco, San Francisco, California, USA and the University of California, Berkeley, Berkeley, California, USA.; Department of Medicine, Stanford University School of Medicine, Stanford, California, USA.; General Counsel for FAMM, Washington, District of Columbia, USA.

**Keywords:** Health policy, Elderly prisoners, Chronic illness, COVID-19, Decarceration, Compassionate release

## Abstract

**Purpose –:**

The purpose of this paper is to provide a historical overview of compassionate release policies in the USA and describe how these policies have been used during the COVID-19 pandemic. The authors then describe how these programs have been shaped by COVID-19 and could be reimagined to address the structural conditions that make prisons potentially life limiting for older adults and those with chronic illness.

**Design/methodology/approach –:**

This paper is primarily descriptive, offering an overview of the history of compassionate release policies before and during the COVID-19 pandemic. The authors augmented this description by surveying state Departments of Corrections about their utilization of compassionate release during 2019 and 2020. The findings from this survey were combined with data collected via Freedom of Information Act Requests sent to state Departments of Corrections about the same topic.

**Findings –:**

The findings demonstrate that while the US federal prison system saw a multifold increase in the number of individuals released under compassionate release policies in 2020 compared to 2019, most US states had modest change, with many states maintaining the same number, or even fewer, releases in 2020 compared with 2019.

**Originality/value –:**

This paper provides both new data and new insight into compassionate release utilization during the COVID-19 pandemic and offers new possibilities for how compassionate release might be considered in the future.

## Aging and compassionate release

Michael Mahoney died alone in a federal prison hospital on July 30, 2004 (Price, 2009). Mr Mahoney had been sentenced to 15years due to a mandatory minimum sentence based on prior convictions. Nine years into his sentence, he was diagnosed with non-Hodgkin’s lymphoma and underwent radiation and chemotherapy. But his disease was aggressive and, ultimately, incurable. He was bedridden and in constant pain. He made a request for compassionate release to allow him to die at home. Despite support from the medical team, the prison warden and his family, the director of the Bureau of Prisons (BOP) refused to petition the court for his release. The judge who sentenced him even reached out, noting his support of the motion. The director of the BOP did not respond, and Mr Mahoney died, behind bars, a few days later (Price, 2009).

Irwin Schiff was 87-years-old and had less than two years left on his sentence (Thompson, 2018). His son Andrew had spent more than two years trying to obtain compassionate release. His request was denied. When Andrew said goodbye to his father at a federal medical facility, Mr Schiff was unconscious and on a respirator but still handcuffed to his hospital bed with an armed correctional officer standing guard nearby (Thompson, 2018).

Carlos Tapia-Ponce had his compassionate release request denied on the grounds of the severity of his crime. He died the next month. Mr Tapia-Ponce was 94-years-old (Thompson, 2018).

Over the past decade alone, US prisons have experienced a 300% increase in people aged 55 or older (Carson and Sabol, 2016). While many of these older adults experience “accelerated aging” (the presence of a greater burden of chronic disease and functional impairment at a younger age than their nonincarcerated peers) (Aday, 2003), compassionate release has not been a mechanism successfully able to alleviate the burden of an aging prison population on either individuals facing geriatric conditions or prisons systems which were not designed with aging, chronic illness or disability in mind (Jefferson-Bullock, 2018). Mandatory minimums, three strikes laws, increase in life without the possibility of parole sentences and enhancements have all served to lengthen prison systems, creating more and more people aging behind bars (Jefferson-Bullock, 2018; Mauer, 2018; Nellis, 2021; Seeds, 2021).

Incarcerating older adults is expensive (Williams et al., 2017) and may no longer fulfill any of the goals of incarceration (Jefferson-Bullock, 2018). Incarceration has typically purported to serve four main purposes: incapacitation, in which an individual is separated from society for the purpose of restricting their ability to commit a crime; deterrence, in which the threat of incarceration prevents future crimes; rehabilitation, which changes an individual’s behavior and thus the likelihood of committing a crime; and retribution, which presumes that an individual who has committed a crime deserves to be given a correspondingly proportionate punishment (Doering, 2020). For older adults, however, these goals are often rendered moot. For one, older adults have the lowest recidivism rates among all age groups, making arguments for incapacitation less relevant (Doering, 2020; Jefferson-Bullock, 2018; Williams et al., 2017). Furthermore, lengthy sentences have been found to be ineffective in deterring crime (Jefferson-Bullock, 2018). In addition, the US prison environment tends to encourage further crime more than rehabilitation, and the rehabilitative programs that do exist are rarely tailored toward the needs of older adults (Jefferson-Bullock, 2018). Finally, the inability of the prison environment to meet the needs of aging populations means that as they age, the quality of life for incarcerated older adults decreases dramatically. This frustrates the retributive goal of incarceration by creating disproportionately harsher sentences for this vulnerable population (Doering, 2020).

The COVID-19 pandemic, which has put incarcerated older adults at undue risk of serious illness and death, has only brought these disparities into starker relief. In this paper, we will describe the history and purpose of compassionate release programs and the way compassionate release has been considered and used during the COVID-19 pandemic. We will consider the ways in which compassionate release programs have or have not been reshaped by the COVID-19 pandemic and reimagine the ways such programs could be used to address the structural conditions of prisons that serve to make the institutions themselves life limiting.

## History of compassionate release

Individuals incarcerated in state and federal prisons, particularly those with indeterminate sentences, have few avenues to petition for early release or resentencing. One avenue, colloquially known as “compassionate release,” relies on medical justifications for release. “Compassionate release” is an umbrella term for programs such as medical parole, elder parole and clemency for medical reasons that serve to release incarcerated individuals facing illness or death. While this term is often used in both academic literature and lay press, very few states use the term in naming their programs. In addition to the federal BOP, only five states (Arizona, Connecticut, Louisiana, South Dakota and Utah) use “compassion” in the title of their policies (Price, 2018). Instead, terms like “medical parole” or “medical release” are more commonly used. FAMM, formerly Families Against Mandatory Minimums, a leading legal and advocacy organization on the criminal legal system, has chosen to use the term compassionate release so “the human experience is foremost in our minds” (Price, 2018, p. 7) as we consider the need for alternatives to incarceration for vulnerable populations.

The history of federal compassionate release is one of shortcomings and stepwise expansions. In 1984, Congress passed the Sentencing Reform Act, which created the US Sentencing Commission and abolished federal parole in an effort to standardize federal sentencing (Berry, 2008). The Act included a provision for early release or resentencing for “extraordinary and compelling reasons.” Federal courts could not entertain compassionate release motions from incarcerated people. Only the federal BOP could file for a reduction in sentence for such individuals. It did so very rarely because it interpreted the criteria for “extraordinary and compelling” circumstances extremely narrowly. Thus, very few motions made it to court to be considered. In 2007, the Sentencing Commission published a set of guidelines to assist judges, which defined eligible circumstances for compassionate release as one or more of the following:

- terminal illness;
- a permanent physical or medical condition;
- deteriorating physical or mental health due to aging which diminishes the ability for self-care within a correctional facility;
- death or incapacitation of the only family member capable of caring for the individual’s child; or
- any other reason deemed “extraordinary and compelling” by the Director of the BOP (Ferri, 2013).

While the BOP recognized there may be other medical reasons that merit consideration for release:

[…] historically motions for compassionate release have been granted only when a prisoner has a terminal illness with a medical prognosis that he or she has one year or less to live (Ferri, 2013, p. 223).

The BOP’s stringent gatekeeping raised significant alarm. In 2013, the Office of the Inspector General (OIG) cited the BOP for its ineffective, inefficient and inconsistent system of compassionate release. While the OIG recommended several policy amendments, these were not heeded by the BOP, and little changed in the way of the underuse of compassionate release. BOP continued as the gatekeeper, preventing courts from considering people who met federal compassionate release criteria. Out of 3,182 individuals requesting compassionate release between 2014 and 2017, for instance, only 306 requests were granted and 81 individuals died while their requests were pending (Jefferson-Bullock, 2018). Finally, in 2018, Congress passed the First Step Act, which, among other things, established that a petitioner who has waited 30 days for their request for compassionate release to be approved by the BOP could file a motion in sentencing court (Doering, 2020). This change empowered sentencing judges to consider and rule on compassionate release motions not brought by the BOP.

Compassionate release in state prison systems has been similarly piecemeal, cumbersome and unavailable. Currently, some type of compassionate release statute exists in 49 states and the District of Columbia (Price, 2018). By and large, states established these policies as a means to reduce correctional costs, with some policies created in the 1980s and 1990s to address the significant number of incarcerated people with HIV/AIDS (Cooper and Bernard, 2020). Yet implementation of these policies remained a challenge in many states. New York State, for instance, passed a compassionate release policy in 1992. But between 1992 and 1998, more individuals died in prison than were released using the compassionate release policy due to overly restrictive eligibility criteria and often delayed processes (Beck, 1999). Wisconsin established a compassionate release policy in 2009, but only eight individuals were released before political and bureaucratic fears prompted the agency responsible for granting compassionate release to tighten the policy in 2011 (Martin, 2019). Surveys of states’ compassionate release policies reveal that these challenges are the norm rather than the exception (Holland et al., 2020; Martin, 2019; Price, 2018). Many state policies were vastly underused due to the numerous steps in the application process, the length of time required in each step, and the subjectivity of the medical eligibility requirements (Martin, 2019). Furthermore, only two states were required to inform incarcerated people about compassionate release policies; very few allowed emergency review, appeal of denial or reapplication; and the majority of states categorically excluded certain individuals from their compassionate release policies due to nonmedical grounds, such as offender categorization, parole eligibility, and minimum sentencing requirements (Cooper and Bernard, 2020; Holland et al., 2020; Price, 2018).

## Public safety and compassionate release

Much of the discourse that surrounds decision-making for compassionate release revolves not on the “extraordinary and compelling” factors that may make a person eligible but around the person’s potential risk to public safety. The consideration of public safety has been integrated into compassionate release policies since their inception. While federal law remits to the court the decision about whether a person who meets medical criteria for compassionate release should, in fact, be freed, the federal BOP usurped that role. Because only the BOP could bring a motion to the court, it used its gatekeeping power to deny compassionate release, relying on its evaluation about public safety by assessing:

[…] the impact of the nature and circumstances of the offense; criminal and personal history and characteristics of the prisoner; the danger, if any, the prisoner poses to the public if released; and the length of the prisoner’s sentence and amount of time left to serve (Fellner and Price, 2012).

Considerations of public safety play a key role in many state programs, as well. As of 2020, 31 state policies had a relative risk requirement in their compassionate release policy which assessed an individual’s risk to society or self. Fifteen states had policies excluding individuals with certain charges – most often sex or violent offenses – from eligibility for compassionate release (Holland et al., 2020).

In a report on compassionate release on the federal level, FAMM and Human Rights Watch describe the BOP’s assessment of public safety concerns, noting that these concerns “can trump all other factors, even for prisoners who are medically eligible, have an acceptable release plan and have no detainers from other jurisdictions pending” (Fellner and Price, 2012). Indeed, public safety factors often prevail as the highest priority within many decision-making spheres. In 32 state jurisdictions, the decision-making power to grant compassionate release lies with parole boards, which are predominantly motivated by public safety concerns (Wylie et al., 2018). This prioritization on both the federal and state level is cemented by decision-makers’ susceptibility to public and political sentiment. A study of public attitudes toward compassionate release found that participants ranked “that the individual was no longer a risk to society” as the most important consideration for release (Wylie et al., 2018). Indeed, studies have shown that many individuals have negative attitudes about the compassionate release of terminally ill incarcerated patients (Boothby and Overduin, 2007) and only slightly agree that people dying in prisons should be treated with compassion (Boothby and Overduin, 2007).

Concerns about public safety are starkly over-inflated relative to actual rates of recidivism for people who may be eligible for compassionate release mechanisms. Compared to the general federal recidivism rate of 41%, the rate of reoffense for adults over 65 years who may be eligible for compassionate release was 13.4% (United States Sentencing Commission, 2017). The recidivism rate of those granted compassionate release was 3.5% (Martin, 2019). In addition, policies that categorically exclude individuals with certain convictions from eligibility for compassionate release do not accurately capture risks to public safety. As Berryessa (2020) describes:

[…] what offenses are classified as “violent” in different jurisdictions are often arbitrary and not indicative of whether a defendant will be a violent risk to the public safety if released; for example, selling drugs within 1,000 feet of a school is considered a violent crime in the state of North Carolina. Yet being convicted of such a crime tells us nothing about whether a person will be a violent threat to the public if released.

Importantly, medical and public safety factors are not evaluated as distinct criteria; in fact, an individual’s risk to public safety is often seen as a function of their health. For example, many policies require that individuals be completely debilitated before they are considered “safe” enough to be eligible for compassionate release, a standard that can be nearly impossible to meet, even for older adults at the end of life. As FAMM and Human Rights Watch describe in their 2012 report, “all too often […] the physical and mental capability to commit a crime is conflated with the likelihood of doing so.” Taken to an extreme, this results in policies such as those in Massachusetts, California, and several other states where individuals released on medical parole may be forced to return to prison if their medical condition improves (Holland et al., 2020; Robinson and Dobrzyn, 2022). These practices reveal a dehumanizing attitude which views incarcerated people as embodiments of risk. They are seen not as dynamic, full beings capable of change and deserving of compassion but as dangerous bodies whose threat is only mitigated by complete physical or mental deterioration. Release within this perspective is motivated less by compassion and more by the hope that the responsibility to punish and detain may be transferred from the state to the individual’s body.

## COVID-19 and compassionate release

The COVID-19 pandemic has had an inordinate impact on individuals incarcerated in prisons and jails. Due to immense overcrowding, a dearth of preventative and medical resources and the high prevalence of comorbidities, incarcerated individuals have become sicker at higher rates and more severely from COVID-19 compared to those in the general population. In the first several months of the pandemic, the COVID-19 case rate for incarcerated individuals was 5.5 times higher than that of the general US population. During these months, the crude death rate from COVID-19 in prisons was 39 deaths per 100,000 individuals, which was higher than the US population rate of 29 deaths per 100,000 (Saloner et al., 2020). Nearly one year later, these outbreaks have led to staggering results. As of April 2021, one in three individuals incarcerated in state prisons and at least 39% of individuals incarcerated in federal prisons have had COVID-19, leading to a total of over 525,000 infections. Over 2,700 individuals have died in custody (Burkhalter et al., 2021).

COVID-19 accelerated the necessity of compassionate release as a “safety valve” (Berry, 2008; Ferri, 2013) for overcrowded prisons. Additionally, unlike other disease outbreaks or structural health challenges that exist within prisons, COVID-19 was not hidden. It was not one media story on one day or several media stories siloed in particular outlets; COVID-19 dominated the national and international landscape for more than a year. While incarceration was not always central to the discussion, the implications of COVID-19 for this population could be easily understood. COVID-19 created an inability to look away, raising further questions about who is incarcerated, for how long and to what end.

The COVID-19 pandemic came on the heels of the First Step Act. Federal compassionate release utilization changed dramatically during this period. The Second Circuit Court of Appeals in USA v. Brooker (Zullo), 976 F.3d 228 (2d Cir. 2020) “freed district courts to consider the full slate of extraordinary and compelling reasons” once individuals were allowed to bring forward motions without the approval of the BOP. As Berry (2008) notes, compassionate release was always intended to be used in circumstances such as severe illness, but most cases were not heard in court. While the BOP interpreted “extraordinary and compelling” to mean situations where the person is terminally ill or close to death, this was always more limited than how the law was written. COVID-19 created new vulnerabilities in this population and in a post-First Step Act era, defendants could now bring that vulnerability to the court’s attention. This allowed, for the first time, for a broader understanding of “extraordinary and compelling” to be considered, and it was. Within the COVID-19 pandemic, individuals were being considered for release due to the risk, rather than the existence, of serious illness.

COVID-19 has fundamentally changed our understanding of extraordinary and compelling, shifting considerations from individual disease prognosis to the potential of prisons themselves to be life limiting. Courts were forced to consider not only an individual’s medical factors but also the overcrowding, lack of resources and other structural inadequacies that put incarcerated individuals at undue risk of serious illness or death. Additionally, COVID-19 shifted how certain considerations were weighted. According to MacDonald (2021), some courts relaxed restrictions on COVID-19 related compassionate release requests: “In United States v. Sherwood, the Sixth Circuit ruled that district courts cannot deny requests for COVID-related compassionate release *solely* on the basis that the inmate remains a danger to the community” (emphasis ours). This challenges practices defined above about how public safety is often weighed against the health of an individual and opens up new possibilities for evaluating the risk and benefits of decarceral decisions.

This court acknowledged that:

[…] the pandemic has made prison sentences deadlier, and we must consider the policy rationale behind keeping sick and elderly prisoners locked up and at risk of death – a sentence not given to them by the courts, but by circumstance (Macdonald, 2021, p. 3).

The shift in compassionate release standards was seen as a “necessary response to a devastating humanitarian crisis” (Macdonald, 2021, p. 4). The Department of Justice came to concede that incarcerated individuals who had one of the conditions listed by the Center for Disease Control as increasing the risk of severe illness from COVID-19 (including cancer, heart disease, lung disease and diabetes) (CDC, 2020) met the medical criteria for Compassionate Release.

While the First Step Act allowed for the possibility of more cases of compassionate release to be brought to the courts, as Roper described:

In the sixteen months following passage of the First Step Act, a relatively modest number of petitions for compassionate release were filed. However, since the start of the COVID-19 pandemic, the filing of compassionate release motions has transformed from a sprinkle of requests into a deluge of frantic and unwieldy findings (29).

In 2020, 2,601 federal cases were approved for release, compared to 145 cases released in 2019 and 24 cases released in 2018 before the First Step Act was implemented (Hymes, 2020; US Sentencing Commission, 2021). The vast majority were filed by incarcerated individuals; the BOP filed only 21 of the winning motions. The health and safety risks created by the pandemic not only “increased the urgency of motions for compassionate release, but have also multiplied the number” of individuals who are filing the requests (Roper, 2020).

Yet, this dramatic shift in approach on the federal level was not mirrored in the states. To better understand how compassionate release was used on the state level during the COVID-19 pandemic, we sent a brief survey (see Departments of Correction Survey) via e-mail to representatives from the Departments of Corrections (DOC) in all 50 US states. Participants were told the goal of the study (approved by the University of California, San Francisco Institutional Review Board) was to understand how various compassionate release programs were used before and during the pandemic. The data we received back was combined with data collected by FAMM via Freedom of Information Act Requests (See Table 1). Of inquiries sent to all 50 states, we did not receive a response or received an email “bounce back” from 21 states. Five states either declined to participate or directed us to their formal research application process. One state asked for more information and agreed to provide their data, but the data has not yet been received. In total, compassionate release information was received from 23 states. This data is difficult to analyze in aggregate. Nine states reported that they did not track information on eligibility for compassionate release programs and/or compassionate release decisions either currently or prior to 2020. This question was not a part of the formal survey but was volunteered in responses. Given the number of states that had not provided data at the time of writing (26) and those that did not explicitly mention what information was tracked, we suspect the total number of states not tracking this information may be higher. Only three states reported new COVID-19 specific release mechanisms in 2020, and those releases were not always tracked separately from other forms of release. Further, as described above, compassionate release is an umbrella term. At least three DOC officials responded to our inquiries asking us to define compassionate release or noting their state does not have a compassionate release policy, despite our survey delineating the specific mechanisms in place in each state. For the purposes of this survey, we asked states to report data on each mechanism that FAMM has classified as being a compassionate release program (Price, 2018).

## Departments of Corrections survey:

1. How many people were eligible for compassionate release programs
  - In 2019:
  - In 2020:
2. How many people applied or were considered for compassionate release programs
  - In 2019:
  - In 2020:
3. How many people were released in 2019 under compassionate release programs?
  - Total:
  - Under (Program A used by the state, i.e. Medical Furlough)
  - Under (Program B used by the state, i.e. Elderly Furlough)
4. How many people were released in 2020 under compassionate release programs?
  - Total:
  - (Program A used by the state, i.e. Medical Furlough)
  - (Program B used by the state, i.e. Elderly Furlough)
  - COVID specific program:
5. If we have more questions, can we reach back out and invite you to participate in an interview? (Yes/No)

These programs are quite diverse. Even among mechanisms with the same or similar names, the criteria for eligibility, the process under which one can be considered for release and the decision-making body can be different. For example, programs designed for the release of individuals with terminal illness range from a prognosis of 30 days or less to live (Kansas) to two years or less to live (Arkansas). Some states have different programs for those with serious medical conditions, those with terminal illness and elderly individuals, while others consider all three categories under the same program. In some states, such as California, medical parole decisions are made by the parole board, while in other states like Massachusetts, the decision is made by the Department Commissioner. In general, programs which include terms like “furlough” or “home confinement” include provisions for the individual to potentially be returned to custody if their medical circumstance improve, though, as described above, this may also be the case under certain medical parole programs. For the purposes of this survey, we did not gather data on the number of individuals returned to prison. Taken together, these factors make it difficult to truly understand if a higher or lower proportion of eligible individuals were released in 2020 compared to 2019.

Four states (Connecticut, Florida, Rhode Island and Wyoming) reported lower number or proportion of releases in 2020 than in 2019. Six states (Alaska, California, Idaho, Iowa, New Jersey and Wisconsin) reported a similar number or proportion of releases in 2020 compared to 2019. However, there is diversity within these; for example, California saw a higher number of released but the same proportion of applications were approved, and Wisconsin reported an increase number of approvals under one program but a decreased number of approvals under another. Eight states (Arizona, Georgia, Kansas, Kentucky, Massachusetts, Minnesota, New Hampshire and North Carolina) reported a higher number of releases in 2020 than in 2019. Again, there was great variability across states. For example, two of these states, Kansas and Kentucky, saw an increase of only one additional release in 2020 compared with 2019, while Minnesota introduced a new COVID medical release program under which 156 individuals were released. One state, New Hampshire, saw more individuals released under At Home Confinement, but one fewer individual was released under medical parole. The data from five states (Illinois, Maine, South Carolina, South Dakota and Tennessee) was offered in such a way that did not allow us to interpret if their number or proportion of releases was higher or lower in 2020 compared to 2019.

Given the limitations on both the available data and our methodology described above, we are hesitant to make a definitive claim on the overall utilization of compassionate release programs during COVID-19 across state DOCs. However, what is clear from this data is that state DOCs as a whole did not have anywhere near the same change in number of releases between 2019 and 2020 that were seen on the federal level. COVID-19 and the First Step Act led to individuals incarcerated in federal prisons to be released at a rate more than 17 fold what had been seen previously, while many states reported numbers of approved releases that were similar to, or at times lower than what had been seen in prior years. Our ongoing data collection, including surveys of legal and policy experts across the states and interviews with key decision-makers in many states, may help gain additional understanding of the landscape of COVID-19 compassionate release across the states. Through this research, we also hope to gain a better understanding of the barriers and facilitators to compassionate release across the states, including those related to discharge planning and the cost and payors of medical care for those released under these programs.

## Reimagining compassionate release as a tool for decarceration

Like many other prisons, the Federal Correctional Institute (FCI) Forrest City Low, in Forrest City, AR, suffered multiple outbreaks of COVID-19 in 2020. Demond Williams, a 37-year-old Black man incarcerated at FCI, experienced the first outbreak in June 2020, with over 500 COVID-19 cases. Mr Williams petitioned for compassionate release, pointing to the BOP’s and FCI Forrest City Low’s inability to adequately implement social distancing measures. Mr Williams also cited a history of smoking, his gender and his race as factors that contribute to his health vulnerability and, thus, as reasons for early release. The motion cited disparities in hospitalization and death based on gender and age, stating that the age-adjusted death rate from COVID-19 at the time was 3.6 times higher for Black individuals than for white individuals. This motion was illustrative of other motions filed during the COVID-19 pandemic, diverging from the historical utilization of the process focusing on an existing terminal or potentially life-limiting illness. Instead, motions like Mr Williams’s pointed toward structural and policy failings of the BOP and the conditions of the federal prison in which a person was incarcerated, as well as medical risk factors and social determinants that increased an incarcerated person’s chances of contracting COVID-19.

Mr Williams’s initial motion in June 2020 was denied. The district court cited a lack of evidence of a compromised immune system that would render him especially susceptible to a severe COVID-19 disease course. However, in October 2020, two changes occurred. First, Mr Williams was diagnosed with hypertension, a risk factor for severe illness due to COVID-19. Second, a current or previous history of smoking was added to the Center for Disease Control’s (CDC) list of risk factors for severe illness due to COVID-19. In light of these two developments, Mr Williams resubmitted a petition for release to the District Court for the Eastern District of Missouri, and on November 24th, 2020, Demond Williams’s motion for release was approved.

The court cited several factors in its decision. The decision noted that Mr Williams had chronic conditions and comorbidities and a history of smoking that heightened his risk of severe illness due to COVID-19 infection, based on CDC guidelines. In its decision, the court also noted Mr Williams’s low risk of recidivism, citing lack of prior criminal history, the accessory nature of the crime related to his sentence and participation in programming and rehabilitation. Finally, the court appealed to the structural dangers of incarceration during the COVID-19 pandemic. They noted the heightened risk of transmission of COVID-19 in carceral settings and the difficulty of social distancing within the FCI buildings. The court determined that the combination of these factors constituted “extraordinary and compelling” reasons to reduce Mr Williams’s sentence and ordered his immediate release.

The implementation of the First Step Act and the sudden emergency of the COVID-19 pandemic led to a fundamental transformation in federal approaches to compassionate release. Prior to the First Step Act, Mr Williams’s case would likely never have made it to court. But, after Mr Williams was able to file his own motion, it was possible for him to be found suitable for release. Further, Mr Williams was released not only because of his own health conditions and prognosis but rather due to the recognition that BOP could not keep him safe given the structural issues inherent to prison settings and the heightened vulnerability to COVID-19 created in the carceral environment. COVID-19 has expanded the scope of compassionate releases to seriously consider the health harming conditions of prisons as a reason for release, beyond individual health status, presenting opportunities for vulnerable patients to be granted the urgent relief they need.

The incarcerated population is sicker and more vulnerable to illness than the general population. A diagnosis of hypertension, a key diagnosis in the decision to release Mr Williams, is not uncommon in prison settings. Individuals incarcerated in jails and prisons in the USA have a higher prevalence of hypertension compared to the general population, with the highest prevalence experienced by Black incarcerated individuals (Binswanger et al., 2009). According to a study in 2009, about 31% of Black individuals incarcerated in 2009 had hypertension along with a higher prevalence of other chronic diseases linked to higher risk of severe COVID-19 (Binswanger et al., 2009; Massoglia and Pridemore, 2015). Further, across the USA, Black Americans, who are vastly overrepresented in prisons, have a shorter lifespan than white Americans, statistics that have only worsened in the last year. During the pandemic, researchers estimate that the reduction in life expectancy was 0.63 years for white individuals, 2.10 years for Black individuals and 3.05 for the Latino population (Andrasfay and Goldman, 2021). The reasons cited for Mr Williams’s release are not rare, nor are they only risk factors for severe COVID-19 illness. Compassionate release, as it has been used by the federal prison system during the pandemic, has the possibility of serving as a true “safety valve” to reduce the structural risks of prisons for incarcerated individuals with chronic illness and advanced age.

Although COVID-19 has highlighted this urgency for compassionate or medical release, it has been clearly evident to people impacted by the carceral system that the conditions of incarceration cannot keep people safe. Structural conditions of prisons, like poor ventilation, overcrowding and inadequate sanitation create an environment ripe for the spread of infectious diseases (McCoy et al., 2020). In 2011, the US Supreme Court ruled that the overcrowding in the California prison system, operating at twice its design capacity, violated incarcerated people’s Eighth Amendment rights and constituted cruel and unusual punishment (Newman and Scott, 2012). For example, in California, a lawsuit was filed when, between 2007 and 2015, the California Department of Corrections and Rehabilitation’s neglect led to a massive Valley Fever epidemic in California prisons located in the Central Valley that led to the death of 53 people and lifelong debilitating health consequences for many others (Klein, 2017).

Additionally, as we grapple as a nation and a global community with the looming threat of climate change, we must consider the disparate impact of extreme weather conditions on incarcerated people; individuals living in prisons have long been subjected to conditions of extreme heat and extreme cold that are only worsening within the reality of climate change. Most prisons are ill-equipped to provide heating or air conditioning in prisons causing illness and death among incarcerated people (Asgarian, 2019; Chammah, 2017; Jones, 2019). Cold temperatures in prisons and jails can be especially fatal when combined with factors like antipsychotic medications that disrupt the body’s ability to regulate temperature, conditions of solitary confinement, poor circulation of air conditioning that concentrates cold air in certain areas, and faulty heating systems (Santo, 2014). Hot temperatures in prisons and jails can be equally lethal with “13 states in the hottest parts of the country lacking universal air conditioning” in carceral settings (Jones, 2019). Specifically, in Texas prisons, only 30% of its prisons have air conditioning, where a high percentage of incarcerated people are vulnerable to illness and death from high temperatures due to antipsychotic medications (21%), hypertension medications (19%), asthma (19%), older age (7% over 55) and diabetes (5%). Extreme temperatures, in addition to conditions like fires, floods and hurricanes, put prisons and their residents at risk, with those who are older and sicker most vulnerable to extreme temperatures, poor air quality and loss of power. It is time to consider how compassionate release could be used to address the structural and environmental conditions that put vulnerable, older adults at risk.

As we imagine new possibilities for compassionate release, several considerations must be at the forefront. First, we must address the lack of available and consistent state-level data on compassionate release and, perhaps, the challenges of the term “compassionate release” itself. As we surveyed state DOCs about their “compassionate release” policies, we confronted the challenges of the disparate systems in place across the country. It is difficult to understand how compassionate release is used across the states when the types of programs and the eligibility for and requirements of those programs vary so widely. It is difficult to fully assess or compare the efficacy of these programs for vulnerable populations when most states do not track how many individuals may be eligible under their programs. Even just the term “compassionate release” created barriers. While our survey specified the exact programs in each state using what was, to our knowledge, the most up-to-date and correct title, the heading of our e-mail and our Institutional Review Board approved materials all used the term “compassionate release.” Initial responses often started with the statement, “There is no compassionate release program” in the state. The programs that we saw as under the umbrella of compassionate release, such as medical parole, were explicitly delineated as separate types of programs. Compassionate release as a term contains both empathy and humanity. It is based on the idea that there is a more benevolent alternative to incarceration for certain groups of people. This is a direct indictment of the lack of compassion built into the system of mass incarceration. It also provides a challenge for systems – or individuals, such as governors – who may not want to be seen as acting with compassion toward individuals convicted of serious crimes. Many state representatives did not want their release programs viewed as compassionate release.

Second, as we look toward expanding and reimagining the role of compassionate release, it becomes imperative to interrogate the concept of public safety as it is used in decisions about compassionate release. Who is considered the public, and whose safety is being prioritized when we consider the fate of older adults? Eligibility for compassionate release is often posed as a balancing act between an incarcerated individual’s medical need and their supposed risk to public safety. Despite the low rates of recidivism demonstrated in this population, older adults and those with chronic illness are still viewed for their potential to commit crimes. This discounts the potential for rehabilitation as well as the role that the individual may play in their family and community outside of prison. Within this calculus, where the individual’s well-being is framed as diametrically opposed to public safety, it becomes clear that the notion of the public was never meant to include incarcerated people or their loved ones. Yet, COVID-19 has demonstrated how linked the health and safety of incarcerated people is to the health and safety of the broader community. Outbreaks of COVID-19 in prisons and jails have had significant repercussions for the health of surrounding communities. As of April 2021, 138,000 correctional officers were infected with COVID-19, of whom 261 died. These outbreaks contributed to half a million additional cases of COVID-19 in the surrounding areas during the early months of the pandemic (Hooks and Sawyer, 2020). Releasing vulnerable older adults from prison could have meant greater health and safety for those living on both sides of prison walls. We call for a reconceptualizing of public safety that considers the well-being of our *entire* community and recognizes that community extends inside and outside of prisons.

Finally, as we imagine new possibilities for compassionate release, we must hold central the wisdom and expertise of our incarcerated elders. People currently and formerly impacted by the criminal legal system have the closest proximity to and deepest knowledge of the problems with mass incarceration and unsafe conditions within prisons. This knowledge is key to creating solutions to our inhumane system of mass incarceration. Partnerships between impacted communities and academics that allow for resource and capacity sharing and center on the leadership of people with lived experiences within the criminal justice system have immense potential for strengthening movements toward policy change for decarceration. More collaboration across impacted communities, academics, policymakers are essential for both better understanding and better using programs of compassionate release.

## Figures and Tables

**Table 1:** Compassionate release across US state prison system in 2019 and 2020

State	State Name for Compassionate Release Program	2019	2020
**Alaska**	Special Medical Parole	Hearings for Special Medical Parole: 0 Hearings for Geriatric Parole: 0 Releases: 0	Hearings for Special Medical Parole: 0 Hearings for Geriatric Parole: 1 Releases: 0
**Arizona**	Executive Clemency Due to Imminent Danger to Death	Requests for Executive Clemency: 12 Denied: 7 Passed away before Governor Action: 3 Released Under Executive Clemency: 2	Requests for Executive Clemency: 9 Denied: 0 Released Under Executive Clemency: 9
**California**	Medical Parole Recall of Sentence Elderly Parole	Hearings for Medical Parole: 34 Medical Parole Requests Granted: 18	Hearings for Medical Parole: 132 Medical Parole Requests Granted: 73
**Connecticut**	Medical Parole Compassionate Parole Release Nursing Home Release	Nursing Home Release Approved: 1 Nursing Home Release Denied: 1	Nursing Home Release Approved: 0
**Florida**	Conditional Medical Release	Granted: 45	Granted: 35
**Georgia**	Medical Reprieve Parole Due to Disability or Advanced Age	Submitted: 87 Approved: 28	Submitted: 97 Approved: 39
**Idaho**	Medical Parole	Granted: 1	Granted: 1
**Illinois**	Executive Clemency for Serious Medical Conditions	Medical Furlough not tracked prior to 2020	Released on Medical Furlough: 26
**Iowa**	No formal compassionate release policies		
**Kansas**	Functional Incapacitation Release Terminal Medical Release	Requested: 0 Approved: 0	Requested: 1 Approved: 1
**Kentucky**	Early Medical Consideration	Denied: 21 Granted: 6 Not Eligible: 3 Passed Away: 7 Pending: 0	Denied: 5 Granted: 7 Not Eligible: 4 Passed Away: 3 Pending: 1
**Maine**	Supervised Community Confinement	January 2017-May 2019: 168 individuals released to Supervised Community Confinement Program	
**Massachusetts**	Medical Parole Executive Clemency-Medical Release	Medical Parole Petitions: 24 Medical Parole Petitions Granted: 4 *Fiscal Year 2019	Medical Parole Petitions: 136 Medical Parole Petitions Granted: 27 *Fiscal Year 2020
**Minnesota**	Conditional Medical Release	Released Under Conditional Medical Release: 6	Released Under Conditional Medical Release: 14 COV1D-19 Conditional Medical Release: Under Final Review: 1 Approved: 158 Denied: 2045 Released: 156
**New Hampshire**	Medical Parole	Released Under At Home Confinement: 43 Released Under Medical Parole: 3	Released Under At Home Confinement: 56 Released Under Medical Parole: 2
**New Jersey**	Medical Parole	Total Requests Received: 8 Not Eligible: 4 Died prior to processing: 2 Granted: 1 Denied: 0 Other: 1	Total Requests Received: 4 Not Eligible: 3 Died prior to processing: 0 Granted: 1 Denied: 0 Other: 0
**North Carolina**	Medical Release Extension of the Limits of Confinement	Considered: 8 Approved: 8	Considered: 15 Approved: 14
**Rhode Island**	Medical Parole	Applied or were considered: 7 Released: 4	Applied or were considered: 5 Released: 3
**South Carolina**	Parole for Terminally Ill, Geriatric, or Permanently Disabled Inmates Parole for Medical Reasons Special Parole of Veterans for Psychiatric Treatment Furlough/Extension of Limits of Confinement	Medical Furlough: Referred: 37 Granted: 3 Medical Parole: Referred: 12 Granted: 1	
**South Dakota**	Compassionate Parole Release		1 individual referred for compassionate parole in 2018 was approved in 2020
**Tennessee**	Medical Furlough Executive Clemency Due to Illness or Disability	Clemency Applications Received (2019-2020): 995 (includes duplicates) Clemency Applications Returned or Denied (2019-2020): 510 Clemency Applications Approved (2019-2020): 0 (remainder still pending)	Clemency Applications Received (2019-2020): 995 (includes duplicates) Clemency Applications Returned or Denied (2019-2020): 510 Clemency Applications Approved (2019-2020): 0 (remainder still pending)
**Wisconsin**	Sentence Modification Due to Extraordinary Health Condition Parole Due to Extraordinary Circumstances (Old-Law Prisoners)	Executive Directive Applicants: 2 Executive Directive Approved: 3 Aged and Extraordinary Health Conditions Petitions: Pending Processing: 0 Not fully processed: 3 Denied: 11 Approved: 1	Executive Directive Applicants: 16 Executive Directive Approved: 0 Aged and Extraordinary Health Conditions Petitions: Pending Processing: 1 Not fully processed: 9 Denied: 102 Approved: 9
**Wyoming**	Medical Parole	Requested: 3 Approved: 0	Requested: 2 Approved: 1
